# Evaluation of viable regions for successful cryopreservation of orchid protocorms

**DOI:** 10.5511/plantbiotechnology.26.0122a

**Published:** 2026-06-25

**Authors:** Tomonari Hirano, Ayaka Yamamoto, Hiroka Miyagi, Soichiro Tsuda, Tomohisa Yukawa, Hisato Kunitake

**Affiliations:** 1 Faculty of Agriculture, University of Miyazaki, Miyazaki, Miyazaki 889-2192, Japan; 2 Tsukuba Botanical Garden, National Museum of Nature and Science, Tsukuba, Ibaraki 305-0005, Japan

**Keywords:** *Bletilla striata*, cryo-plate, shoot apical meristem, vitrification

## Abstract

In Orchidaceae, the protocorm, which forms after seed germination, serves as a crucial genetic resource for plant preservation and breeding. To establish a long-term conservation method for the protocorm, in this study, we developed cryopreservation methods. Protocorms of *Bletilla striata*, a terrestrial orchid species, were precultured for 3 days in a medium containing 0.3 M sucrose and then cryopreserved using vitrification. Although viable cell staining indicated the presence of many surviving cells in the protocorms, the regrowth rate was extremely low. We categorized the stained regions based on the presence or absence of staining in the shoot apical meristem (SAM). The staining rate in the SAM region was low after cryopreservation, which was considered the cause of the low regrowth rate. To enable cryopreservation of the protocorms, the V-Cryo-plate method was adopted, and the treatment times for the solutions used in dehydration were investigated. Treatment times of 45 min and 2 h for the loading and vitrification solutions were found to be appropriate, respectively. Furthermore, an investigation of the effect of adding plant growth regulators to the preculture medium showed that adding 0.1 µM 1-naphthalene acetic acid resulted in a high SAM survival rate of 88% and regrowth rate of 87%, comparable to that observed with survival cell staining. Therefore, survival in the SAM region is important for regrowth in cryopreserved protocorms. Confirmation of survival regions is an important indicator for bridging the gap between the survival and regrowth rates.

## Introduction

Orchidaceae is one of the largest families of flowering plants and includes approximately 28,000 species with distinctive physiological and ecological characteristics (Chase et al. 2015; Christenhusz and Byng 2016). One characteristic of this family is the germination process from the seed (Yeung 2017). The embryos of orchid species are not well developed and do not give rise to a cotyledon; however, one of the temperate terrestrial species, *Bletilla striata*, develops a rudimentary cotyledon in the embryo. Upon seed germination, the embryo differentiates a shoot apical meristem (SAM) and forms a structure called a protocorm, which aids in establishing symbiosis with fungi necessary for germination. While artificially propagating orchid plants from seeds, in vitro asymbiotic culture is often preferred. Protocorms also form during culture, and in some cases, protocorm-like bodies (PLBs), similar to embryogenic calli, proliferate from the protocorm. Seeds, protocorms, and PLBs are important materials for the conservation of genetic resources and their utilization in breeding.

There are many endangered species in Orchidaceae, and cryopreservation has been studied as a long-term conservation method for the species. Immature and mature seeds, protocorms, PLBs, and more have been successfully cryopreserved as orchid plant materials for long-term conservation (reviewed in Das et al. 2021; Hirano et al. 2006; Popova et al. 2016). In *B. striata*, cryopreservation has been performed on immature and mature seeds, embryos, and protocorms during in vitro asymbiotic culture (Hirano and Mii 2007; Hirano et al. 2005a; Ishikawa et al. 1997; Jitsopakul et al. 2008). Although relatively high survival rates were obtained after cryopreservation in the early culture stages, the survival rates after cryopreservation decreased as the days progressed. No viable individuals were obtained in protocorms cultured for 12 days (Jitsopakul et al. 2008). Since the degree of dehydration tolerance varies depending on the plant materials used for the cryopreservation, it is necessary to consider methods specific to each material. Difficulty in the cryopreservation of protocorms has also been reported in other species (Nikishina et al. 2007; Pimda and Bunnag 2010; Soonthornkalump et al. 2019; Sopalun et al. 2010). Differences in protocorm development among species must also be considered.

One of the cryopreservation methods used for orchids is vitrification, in which explants are osmotically dehydrated by exposing them to a highly concentrated vitrification solution, and three treatments are generally performed before immersing the explants in liquid nitrogen (Sakai and Engelmann 2007). Vitrification has been shown to have wide applicability in orchids (Hirano et al. 2011). Before dehydration by the vitrification solution, the explants or mother plants are cultured on sucrose-enriched medium to ensure a high level of survival and recovery growth after cryopreservation. For example, in orchids, preculturing immature seeds on solidified medium supplemented with 0.3 M sucrose for 3 days was found to improve dehydration tolerance and germination (Hirano et al. 2005a, b). The explants are then treated with loading solution (LS) consisting of 2 and 0.4 M glycerol and sucrose, respectively, with a moderate concentration to prepare them for exposure to the vitrification solution; LS has been reported to be highly effective for inducing dehydration tolerance to the vitrification solution (Matsumoto et al. 1994; Nishizawa et al. 1993). Finally, osmotic dehydration using the vitrification solution is performed to remove a major part of the freezable cell water. PVS2 (Sakai et al. 1990) is a vitrification solution widely used for plants, composed of 30% (w/v) glycerol, 15% (w/v) ethylene glycol, 15% (w/v) DMSO, and high-concentration sucrose (0.4 M). As an improved method based on the vitrification method, the V-Cryo-plate method, which uses an aluminum cryo-plate for vitrification, has been developed (Yamamoto et al. 2011, 2012). This method involves embedding small samples, such as shoot tips, in sodium alginate beads within the wells of a cryo-plate, then subjecting the entire plate to solution treatment using the vitrification method. The V-Cryo-plate method has the advantages of enabling easy solution processing without damaging the sample and allowing rapid sample cooling and heating.

In this study, we attempted cryopreservation of developed *B. striata* protocorms. In *B. striata*, seed germination and subsequent protocorm development under asymbiotic culture conditions are stable and uniform, making it extremely useful for protocorm research. As several meristematic tissues exist in the PLBs, regrowth is possible if any of them survive after cryopreservation. However, for protocorms, the survival region must contain a SAM, and consideration of the viable region is important. It is crucial to confirm regrowth by culturing protocorms after cryopreservation. Furthermore, if changes in protocorms can be visualized as the viable region during the evaluation of experimental methods and processes, it becomes possible to identify optimal conditions more efficiently. As no studies have yet been conducted considering the survival region of protocorms, we aim to establish a cryopreservation method for protocorms with an evaluation focused on the viable region. As mentioned above, cryopreservation of the developed *B. striata* protocorms is difficult, and overcoming this issue requires inducing dehydration tolerance and improving the dehydration process. We investigated the pre-culture medium as a means to induce dehydration tolerance and examined the LS and vitrification solution treatment times as ways to improve the dehydration process.

## Materials and methods

### Plant materials

Mature seeds of *Bletilla striata* Rchb.f. were stored with silica gel at 4°C. The seeds were surface-sterilized with a sodium hypochlorite solution (1% available chlorine) containing 0.1% polyoxyethylene (20) sorbitan monolaurate for 10 min and then washed 3 times with sterilized water. The seeds were sown on basal medium consisting of 0.2% gellan gum-solidified New Dogashima (ND) medium (Tokuhara and Mii 1993) with 88 mM sucrose and cultured at 25°C with a 16 h light/8 h dark cycle for 9 days.

As a preculture, the protocorms cultured for 9 days were transferred to 0.2% gellan gum-solidified ND medium containing 0.3 M sucrose with/without plant growth regulators (PGRs): 0.1–10 µM 1-naphthalene acetic acid (NAA), 0.1–10 µM 6-benzylaminopurine (BAP), 0.1–10 µM gibberellin A3 (GA_3_), and 0.1–10 µM abscisic acid (ABA). The protocorms were cultured at 25°C with a 16 h light/8 h dark cycle for 3 days.

### Cryopreservation

The cryopreservation procedure for vitrification was performed according to Hirano et al. (2005a). Briefly, the precultured protocorms were treated with LS in cryotubes for 15 min at 25°C. The LS was replaced with 2 ml of vitrification solution, PVS2, and the samples were treated for 2 h at 0°C. Then, the cryotubes were directly plunged into liquid nitrogen (LN) for at least 30 min. The cryotubes were then rapidly warmed at 38°C. PVS2 was drained from the cryotubes and 1 ml of liquid ND medium supplemented with 1.2 M sucrose was added to the cryotubes. After 10 min of treatment, liquid ND medium supplemented with 0.4 M sucrose was added in duplicate at 0.5-ml intervals every 10 min. The treated protocorms were cultured on basal medium at 25°C with a 16 h light/8 h dark cycle.

A cryo-plate with 12 wells (001-001J, Taiyo Nippon Sanso Corp., Japan) was used for the V-Cryo-plate method; one precultured protocorm was transferred to each well filled with 2% (w/v) sodium alginate in calcium-free ND basal medium (2 µl). The wells were covered with 0.1 M calcium chloride solution and allowed to polymerize for 15 min. After removing the calcium chloride solution, the plates were placed in the cryotubes containing 2.0 ml of LS and immersed for 15 to 60 min at 25°C. Subsequently, they were placed in cryotubes containing 2.0 ml of PVS2 and immersed for 1 to 6 h at 0°C. The plates were transferred to cryotubes filled with LN, and the entire tube was plunged into LN for at least 30 min. The plates were immersed for 2 min in 20 ml of 1.2 M sucrose solution heated to 38°C. The treatments, after warming, were performed in the same manner as vitrification. The treated protocorms were cultured on basal medium. The alginate beads containing the protocorms were placed on the basal medium at 25°C with a 16 h light/8 h dark cycle.

### Evaluation of viable region and regrowth

**Figure 1:**
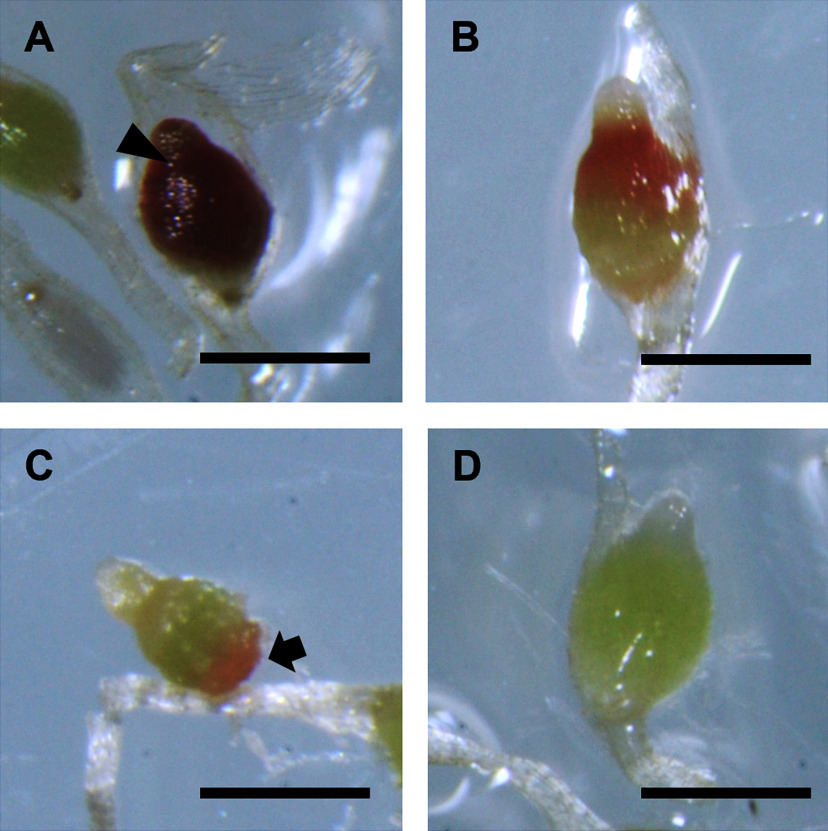
Figure 1. Protocorm categories based on TTC stained region. (A) SAM region with more than half of the protocorm stained. The arrowhead indicates the SAM region at the base of the cotyledon. (B) SAM region with less than half of the protocorm stained. (C) SAM region was not stained, but part of the protocorm was stained. The arrow indicates the stained region at the basal side of the protocorm. (D) Unstained protocorm. Bars=0.5 mm.

Viable regions in the protocorms were estimated via the 2,3,5-triphenyltetrazolium chloride (TTC) stainability test. The cryopreserved protocorms were cultured on the basal medium for 1 day, then incubated in 1% (w/v) TTC solution for 1 day at 25°C in the dark. Protocorms that were stained even partially were considered “stained”, and the total TTC stainability was calculated. Furthermore, the stained regions were classified into three categories based on the presence or absence of SAM staining, SAM (+) or SAM (−), respectively, and whether they were below or above half the protocorms (Figure 1), and the proportion of plants in each category was calculated. Protocorm regrowth was observed 4 weeks after culture following cryopreservation.

### Statistical analysis

Tukey’s test was used to compare the values of TTC stainability in each category. The Regrowth rates of protocorms after cryopreservation by the vitrification method and the V-Cryo-plate method were compared using a *t*-test. The differences were considered significant at the 5% level.

## Results and discussion

### Cryopreservation of protocorms by vitrification

**Figure 2:**
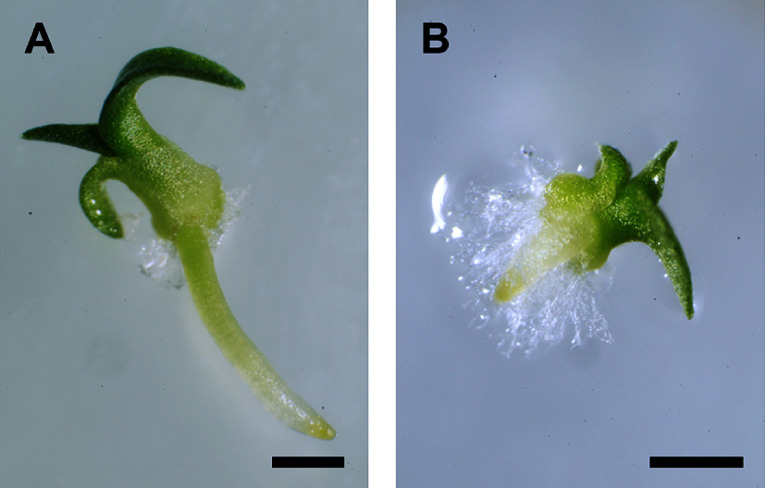
Figure 2. Regrowth of cryopreserved protocorms by vitrification after 4 weeks of culture. Untreated control (A), regrown protocorms after cryopreservation (B). Bars=1 mm.

To investigate effective cryopreservation methods for protocorms after 9 days of culture, we first attempted cryopreservation using the vitrification method previously developed for immature and mature embryos of *B. striata* (Hirano et al. 2005a; Ishikawa et al. 1997). The protocorms were precultured for 3 days on the medium supplemented with 0.3 M sucrose, then treated with LS for 15 min and PVS2 for 2 h before cryopreservation. Total TTC stainability was 87%, but the regrowth rate was significantly lower, at 17% (Table 1). The TTC-stained regions in the protocorms were classified into three patterns: staining of more than half the protocorm including the SAM region (Figure 1A), staining of less than half the protocorm including the SAM region (Figure 1B), and staining of a portion of the protocorm excluding the SAM region (Figure 1C). When TTC stainabilities were calculated for each category, it became evident that the SAM region in the protocorms exhibited lower stainability (Table 1). In regrown individuals, reflecting the TTC-stained region, growth of the basal region in the protocorm or plantlets was suppressed, and rooting was observed from the region closer to the SAM (Figure 2). No PLB formation was observed. The morphology of the regrown individuals suggested that the survival of the SAM is crucial for vigorous regrowth.

**Table 1:** Table 1. TTC stainability and regeneration of the cryopreserved protocorms by vitrification.

Total TTC stainability (%)	TTC stainability of each category (%)			Rregrowth rate (%)
	SAM (−): Part of Protocorm	SAM (+)		
		Less than half of protocorm	Half of protocorm or more	
87.3±2.4	57.7±6.4	15.1±2.2	14.5±1.9	16.9±4.9

Data represent mean of three replicates±standard error.

In protocorms, enlargement and vacuolization of basal cells are commonly observed during growth (Yeung 2017). Similarly, in *B. striata*, vacuoles were particularly well developed in the basal cells of the protocorms (Supplementary Figure S1). The staining pattern reflects the difficulty of cryopreservation for each tissue or region. Vacuolized cells are thought to be difficult to sufficiently dehydrate to freezable water and induce dehydration tolerance.

### Cryopreservation of protocorms by V-Cryo-plate method

Compared with the vitrification method, the V-Cryo-plate method had much higher sample cooling and heating rates. This high rate makes intracellular freezing less likely, leading to expectations of higher regrowth rates. Therefore, the V-Cryo-plate method was applied to the protocorms. However, when using the same preculture conditions, LS treatment time, and PVS2 treatment time as those used in the vitrification method, no improvement in TTC stainability was observed (Table 2). As no improvement in TTC stainability was observed by changing the technique alone, the dehydration tolerance of the protocorms must be enhanced. Using the V-Cryo-plate method, we investigated the effects of LS and PVS2 treatment time on survival. When the LS exposure time was set to 15 min and PVS2 exposure time was varied from 1 to 6 h, no significant difference in TTC stainability was observed (Table 2). Regarding TTC stainability by region, no significant difference was observed for protocorms with stained SAM regions. However, for the stained protocorms without stained SAM regions, TTC stainability significantly increased with exposure times of 2 h or longer compared with that at 1 h. Subsequent experiments were conducted using a PVS2 exposure time of 2 h. When the LS exposure time was varied from 15 to 60 min, the proportion of the protocorm with a stained SAM region increased as the exposure time increased. The proportion of protocorms stained by half or more part including the SAM region reached the highest value of 40% with a 45-min treatment time (Table 3).

**Table 2:** Table 2. Effects of PVS2 exposure time on TTC stainability of protocorms after cryopreservation by V-cryo plate method.

PVS2 exposure time (h)	Total TTC stainability (%)	TTC stainability of each category (%)		
		SAM (−): Part of protocorm	SAM (+)	
			Less than half of protocorm	Half of protocorm or more
1	68.5±4.8^NS^	39.7±4.5^b^	12.1±2.6^NS^	16.7±5.8^NS^
2	80.6±4.1^NS^	63.4±2.3^a^	13.3±2.4^NS^	3.9±1.9^NS^
4	81.9±3.3^NS^	77.6±3.3^a^	1.9±1.0^NS^	2.4±0.5^NS^
6	81.8±8.1^NS^	70.8±4.2^a^	7.5±4.6^NS^	3.5±2.1^NS^

The protocorms were treated with LS for 15 min. Data represent mean of three replicates±standard error. Different letters in each column are significantly different at the 0.05 level by Tukey’s test (*n*=3). NS: there were no significant differences (*p*≥0.05) between the values in each column.

**Table 3:** Table 3. Effects of LS exposure time on TTC stainability of protocorms after cryopreservation by V-cryo plate method.

LS exposure time (min)	Total TTC stainability (%)	TTC stainability of each category (%)		
		SAM (−): Part of protocorm	SAM (+)	
			Less than half of protocorm	Half of protocorm or more
15	92.7±2.5^NS^	67.0±4.4^a^	8.6±2.1^b^	17.1±3.2^b^
30	94.2±2.3^NS^	54.3±4.8^a^	32.0±4.2^a^	8.0±1.4^b^
45	89.7±3.4^NS^	20.0±3.1^c^	30.2±2.4^a^	39.6±2.7^a^
60	93.9±0.6^NS^	35.3±5.1^b^	24.8±4.0^a^	33.9±9.5^a^

The protocorms were treated with PVS2 for 2 h. Data represent mean of three replicates±standard error. Different letters in each column are significantly different at the 0.05 level by Tukey’s test (*n*=3). NS: there were no significant differences (*p*≥0.05) between the values in the column.

In the PLBs of *Dendrobium nobile*, severe plasmolysis and chromatin condensation, and vesicles encapsulated with membranous material in the cytoplasm were observed following PVS2 dehydration treatment (Jiang et al. 2019). Therefore, vitrification treatment induces chemical toxicity and osmotic stress in the cells, leading to changes associated with programmed cell death. Pretreatment with LS containing cryoprotectants at a lower concentration than that of the vitrification solution is highly effective for inducing dehydration tolerance to the vitrification solution (Matsumoto et al. 1994; Nishizawa et al. 1993). Compared with the 15–20-min LS treatment time commonly used, the extended treatment time used in this study likely conferred sufficient dehydration tolerance to PVS2 treatment, particularly in the SAM region. In PLBs, extending the LS treatment time to 60–80 min also improved survival rates (Mohanty et al. 2012, 2013; Yin and Hong 2009). It is necessary to determine the treatment time based on factors such as the developmental stage and size of the plant material, degree of vacuolization mentioned above, and time required for solution penetration.

### Effects of PGRs added to preculture medium

To improve survival around the SAM, representative PGRs were added to the preculture medium, and their effects were investigated. Each PGR was added at concentrations ranging from 0.1 to 10 µM, and TTC stainability was compared (Table 4). At 0.1 µM NAA, the percentage of protocorms stained by half and more than half was significantly higher, at 67%, than that of the preculture medium without PGRs, and it was revealed that most of the TTC-stained region contained the SAM (88%). In contrast, no improvement was observed at any concentration for ABA, BAP, or GA_3_. At 10 µM ABA, a decrease in protocorms stained with the SAM region was observed. Based on these results, the addition of PGRs to the preculture medium for the protocorms revealed that 0.1 µM NAA addition is optimal.

**Table 4:** Table 4. Effects of plant growth regulators added to preculture medium on TTC stainability of protocorms after cryopreservation by V-cryo plate method.

Plant growth regulators	Concentration (μM)	Total TTC stainability (%)	TTC stainability of each category (%)		
			SAM (−): Part of protocorm	SAM (+)	
				Less than half of protocorm	Half of protocorm or more
ABA	0	89.7±3.1^NS^	20.0±2.4^b^	30.2±2.4^a^	39.6±2.7^a^
	0.1	86.0±3.5^NS^	10.0±2.9^b^	22.5±3.1^ab^	53.5±7.9^a^
	1	93.4±0.6^NS^	20.9±7.7^b^	29.5±4.4^a^	43.0±4.1^a^
	10	89.1±2.9^NS^	65.7±4.9^a^	13.2±2.4^b^	10.2±1.2^b^
BAP	0	73.8±5.7^NS^	16.3±2.4^NS^	17.7±4.5^NS^	39.8±6.5^NS^
	0.1	62.6±17.0^NS^	13.0±3.3^NS^	15.5±1.7^NS^	34.1±15.1^NS^
	1	70.0±5.6^NS^	22.6±3.4^NS^	14.4±1.3^NS^	33.0±2.9^NS^
	10	71.0±4.1^NS^	15.1±1.6^NS^	20.6±2.0^NS^	35.3±7.0^NS^
GA_3_	0	69.6±2.8^NS^	11.4±3.0^NS^	12.7±4.3^NS^	45.4±3.7^a^
	0.1	70.8±8.1^NS^	17.9±2.6^NS^	22.5±3.9^NS^	30.4±4.0^ab^
	1	65.6±4.8^NS^	20.7±1.9^NS^	17.0±3.9^NS^	27.8±3.0^b^
	10	69.5±5.6^NS^	13.7±2.0^NS^	20.3±4.1^NS^	35.4±3.0^ab^
NAA	0	92.6±3.2^NS^	37.6±11.9^a^	32.5±13.2^NS^	22.9±12.6^b^
	0.1	97.9±1.4^NS^	9.5±1.1^b^	21.0±4.4^NS^	67.4±6.6^a^
	1	97.0±0.7^NS^	18.9±7.2^ab^	28.6±6.0^NS^	49.5±13.0^ab^
	10	93.2±1.0^NS^	22.7±6.4^ab^	34.1±0.8^NS^	36.4±6.6^b^

The protocorms were treated with LS for 45 min and PVS2 for 2 h. Data represent mean of three replicates±standard error. Different letters in each column at each plant growth regulator are significantly different at the 0.05 level by Tukey’s test (*n*=3). NS: there were no significant differences (*p*≥0.05) between the values in each column at each plant growth regulator.

In this experiment, the addition of 0.1 µM NAA was the most effective in improving the viable region after cryopreservation, suggesting that NAA enhances dehydration tolerance in protocorm cells across a broad region, including the SAM. Auxin is a key hormone in mediating plant responses to abiotic stresses, either ABA-dependently or independently (Musazade et al. 2025). In the protocorms of *B. striata*, no improvement in survival after cryopreservation was observed, even with exogenous ABA addition, suggesting that ABA-independent pathways may primarily mediate the improvement in dehydration tolerance in the NAA-added preculture medium. Previous studies have reported improved survival rates after cryopreservation in orchid tissues or organs when exogenous ABA was applied during preculture (Reviewed in Hirano et al. 2006). The use of PGRs appropriate for each plant material or developmental stage is necessary, and further research regarding the enhancement of drought tolerance by NAA in the protocorms of *B. striata* is required.

### Regrowth of protocorms after cryopreservation by V-Cryo-plate method

**Figure 3:**
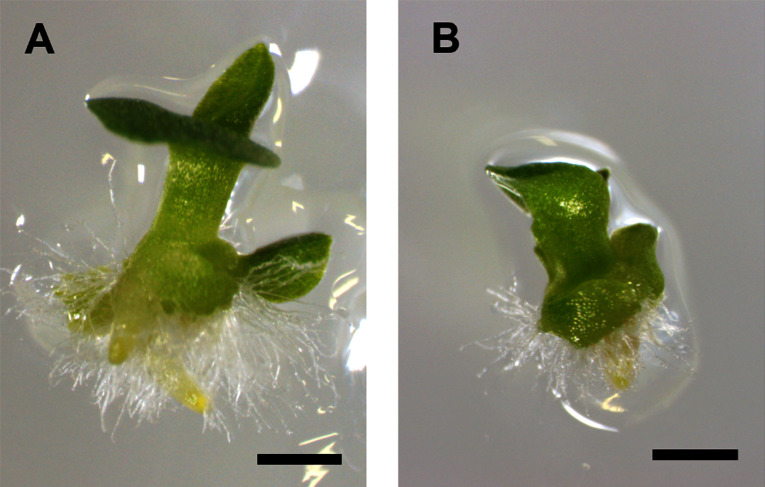
Figure 3. Regrowth of cryopreserved protocorms by V-Cryo-plate method after 4 weeks of culture. Protocorms treated by V-Cryo-plate method without (A) or with (B) immersion into LN. Bars=1 mm.

Preculturing in 0.1 µM NAA-supplemented medium followed by prolonged LS treatment resulted in significantly higher TTC stainability in the SAM region after cryopreservation. Regrowth was confirmed after 4 weeks of culture for protocorms treated under the improved conditions with or without immersion in LN (Figure 3). In both protocorms, growth inhibition was observed in the basal region of the protocorms, similar to the vitrification method. However, the regrowth rate after cryopreservation was significantly higher, at 87.8±7.7 (mean±standard error), compared with that after the initial vitrification method. This is primarily due to improved dehydration tolerance prior to PVS2 treatment, rather than differences between the vitrification and V-Cryo-plate methods.

The seeds of *B. striata* are suitable for protocorm research because they exhibit uniform germination and subsequent development under asymbiotic culture conditions. In this study, protocorms were used as a model to analyze the viable region after cryopreservation. Confirmation of viable regions using methods such as TTC staining is important for bridging the gap between survival and regrowth rates. To improve cryopreservation methods for plants, it is recommended to also confirm the viable region in order to assess the induction status of dehydration tolerance.

## Abbreviations

ABA: abscisic acid
BAP: 6-benzylaminopurine
GA_3_: gibberellin A3
LN: liquid nitrogen
LS: loading solution
NAA: 1-naphthalene acetic acid
ND: New Dogashima
PGR: plant growth regulator
PLB: protocorm-like body
SAM: shoot apical meristem
TTC: 2,3,5-triphenyltetrazolium chloride

## Description of supplementary files

Supplementary Figure S1. Histological observation of zygotic embryo and protocorm.
