# Supplementary material for: Evaluation of viable regions for successful cryopreservation of orchid protocorms
Source: Plant Biotechnol (Tokyo). 2026 Jun 25;43(2):175–80. doi: 10.5511/plantbiotechnology.26.0122a (PMC13324209; doi:10.5511/plantbiotechnology.26.0122a)
Supplement: Supplementary Data [file plantbiotechnology-43-2-26.0122a-s001.pdf]

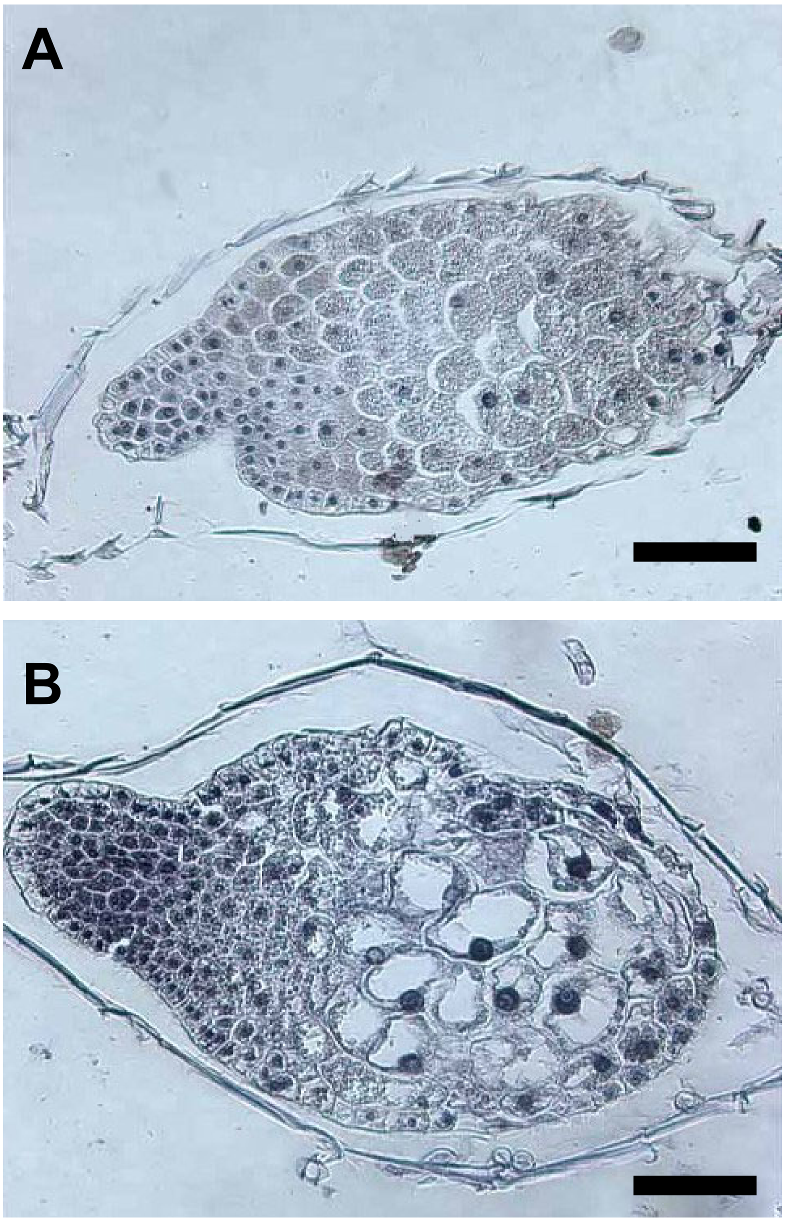

Supplementary Figure S1. Histological observation of zygotic embryo and protocorm in *B. striata*. The zygotic embryo (A) and protocorm (B) were formed in the asymbiotic seed culture on the basal medium for 6 and 9 days, respectively.
Bars = 0.1 mm.
